# Review: the effect of light on the key pigment compounds of photosensitive etiolated tea plant

**DOI:** 10.1186/s40529-021-00329-2

**Published:** 2021-12-11

**Authors:** Cuinan Yue, Zhihui Wang, Puxiang Yang

**Affiliations:** 1 Jiangxi Sericulture and Tea Research Institute, Nanchang, 330043 China; 2 Jiangxi Key Laboratory of Tea Quality and Safety Control, Nanchang, 330203 China

**Keywords:** Light, Photosensitive etiolated tea plants, Key pigment compounds, Chlorophyll, Carotenoids

## Abstract

**Background:**

Light is the ultimate energy source of plant photosynthesis, which has an important impact on the growth, development, physiology and biochemistry of tea plant. Photosensitive etiolated tea plant belongs to a kind of colored leaf plant, which is a physiological response to light intensity. Compared with conventional green bud and leaf of tea plant, the accumulation of pigment compounds (chlorophyll and carotenoids, etc.) closely related to a series of reactions of photosynthesis in photosensitive etiolated tea plant is reduced, resulting in the difference of leaf color of tea. This specific tea resource has high application value, among which high amino acid is one of its advantages. It can be used to process high-quality green tea with delicious taste and attractive aroma, which has been widely attention. The mechanism of the color presentation of the etiolated mutant tea leaves has been given a high topic and attention, especially, what changes have taken place in the pigment compounds of tea leaves caused by light, which makes the leaves so yellow. At present, there have been a lot of research and reports.

**Purpose of the review:**

We describe the metabolism and differential accumulation of key pigment compounds affecting the leaf color of photosensitive etiolated tea that are triggered by light, and discuss the different metabolism and key regulatory sites of these pigments in different light environments in order to understand the “discoloration” matrix and mechanism of etiolated tea resources, answer the scientific question between leaf color and light. It provides an important strategy for artificial intervention of discoloration of colored tea plant.

**Conclusion:**

The differential accumulation of pigment compounds in tea plant can be induced phytochrome in response to the change of light signal. The synthesis of chlorophyll in photoetiolated tea plants is hindered by strong light, among which, the sites regulated by coproporphyrinogen III oxidase and chlorophyllide a oxidase is sensitive to light and can be inhibited by strong light, resulting in the aggravation of leaf etiolation. The phenomenon can be disappeared or weakened by shading or reducing light intensity, and the leaf color is greenish, but the increase of chlorophyll-b accumulation is more than that of chlorophyll-*a*. The synthesis of carotenoids is inhibited strong light, and high the accumulation of carotenoids is reduced by shading. Most of the genes regulating carotenoids are up-regulated by moderate shading and down-regulated by excessive shading. Therefore, the accumulation of these two types of pigments in photosensitive etiolated tea plants is closely related to the light environment, and the leaf color phenotype shape of photosensitive etiolated tea plants can be changed by different light conditions, which provides an important strategy for the production and management of tea plant.

## Introduction

Tea (*Camellia sinensis*) plant is a widely planted perennial woody cash crop, which can be used to process non-alcoholic beverages with consumers friendly (Zeng et al. 2018). Generally, plant leaves are green, however, in the process of plant evolution, in the face of a variety of complex ecological environment, plants will evolve a variety of complex mechanisms to respond to environmental stress to adapt to the environment, and leaf color variation is one of the strategies (Xu et al. 2021). Tea plant is a perennial green leafed plant, however, there are also mutants of leaf color, such as etiolated, albino and purple, and, their buds, leaves and tender stems are yellow, white and purple. The buds and leaves of etiolated and albino contain higher levels amino acid than green’s, which has attracted the attention and favor of the market and producers (Shin et al. 2018; Song et al. 2017). Etiolated, albino and purple tea plants are considered as high-quality breeding materials. Under specific environmental conditions, the pigments accumulation mechanisms in these tea plants are different, which makes the leaves show yellow or white or purple or mixed colors. According to the different inducing factors, they can be divided into temperature sensitive and light sensitive tea resources. Most of the etiolated tea plants belong to photosensitive type. Under high light intensity, the leaves are yellow, while are shaded, the leaves turned green or the degree of etiolated decreased. The yellow variation of tea leaves is relatively stable, for example, the yellow tender shoots of “Huangjinya” can last for three seasons (summer, autumn and next spring). This phenotype is fascinating, therefore, the problem of etiolated of tea leaves has been widely studied. Among them, the gene expression profile, genetic structure, some specific base mutation sites and differential accumulation of secondary metabolites of photosensitive etiolated tea plants have been gradually clarified. Especially in different light environments, the synthesis and regulation of these pigment compounds have been focused. These studies can effectively explain why the leaf color of photoetiolated tea plants is so yellow. In this review, we focus on the effects of light on the synthesis and differential accumulation of pigments in photoetiolated tea plants, with chlorophyll and carotenoids as the main objects of discussion. This paper summarizes the research progress on the effects of light on the key compounds of leaf color phenotype of photosensitive etiolated tea plant, and comprehensively generalizes the leaf color regulation mechanism in response to light signals in tea plants, so as to deeply and systematically understand the discoloration mechanism of photosensitive etiolated tea resources, and answer the scientific question of why tea leaves are so yellow. It provides a theoretical basis for taking reasonable light control measures to make full use of characteristics of etiolated tea varieties and improve the biomass.

### Effect of light on the growth and development of tea plant

Plants use complex photoreceptors and signal systems to continuously monitor external light irradiation parameters, and finally form complex pathway information to regulate their physiological and developmental aspects (Argüello-Astorga et al. 1998). Light is also a key environmental factor to regulate the growth and development of plants, and which is not only an essential energy for plants, but also an important signal to affect the transition of plant seedlings from etiolation to de-etiolation. It can participate in the regulation of plants in the form of photoperiod, light intensity and light quality, and it is also a stimulator for plant growth and development, morphogenesis and physiological metabolism, biosynthesis of cell components and gene expression in the whole life cycle of plants. Especially, light quality has a great impact on photosynthesis, growth, quality and yield of plants (Anna et al. 2001; Qian et al. 2009). During plant growth, the biological characters such as plant height, stem diameter, leaf structure, leaf area and leaf color are significantly changed by reducing light intensity, and the changes of photosynthetic pigment content and photosynthetic efficiency are also affected. Different light quality has different effects on plant growth and development. Red light plays an important role in the development of photosynthetic system and can increase starch accumulation by inhibiting photosynthetic translocation; blue light has vital effects on chloroplast development, chlorophyll formation and stomatal opening (Sæbø 1995; Senger 2008). The agronomic traits, physiological and biochemical characteristics and internal structure of tea plants are easily affected by light. For example, tea plant growing under full light will have small leaf shape, thick leaves, short internodes and hard and brittle leaves due to strong light. Under the shade, tea plant has large leaves, thin leaves, long internodes and soft leaves. When the tea plant is moderately shaded, the physical and chemical components in the tea plant are also changed. After shading, the contents of nitrogen-containing compounds such as caffeine and amino acids are increased, while the carbon metabolites such as tea polyphenols, crude fiber and carbohydrates are decreased. Proper shading can reduce the thickness of wax layer and palisade tissue, and the arrangement of sponge tissue is more loosely (Pan 1965; Wan 2008). Light has an important effect on plant leaves before and after in vitro. Different LED monochromatic are carried for pre-harvest and pro-harvest the fresh leaves of tea plant to improve flavor of tea (Ai et al. 2017; Lee et al. 2013). Light is particularly important for the physiological development of leaves of photosensitive etiolated tea plant, under high light intensity, the grana of chlorotic tea plant that “Baijiguan” is scarce, and the structure and interval of thylakoid membrane system are destroyed (Wu et al. 2016). The mechanism of tea leaves responding to light signals is induced to this kind of “internal injury”, and the substrates regulating the synthesis of pigments are affected, with the synthesis and accumulation of pigments differentiated, as well as tea leaf color is induced. This process is regulated by a series of light responsive receptors and related genes and enzymes. This procedure makes the scientific phenomenon of leaf discoloration complicated and rigorous, but also interesting.

### Photosensitive signals in higher plants

The developmental plasticity of light signal in plants is endowed by special information transduction photoreceptors, and plants can sense, receive and transmit light signals through different photoreceptors, and then regulate the working mechanism of corresponding genetic media, so they can complete the corresponding series of reactions under different light signals. The main photoreceptors of higher plants are five phytochromes, two cryptochrome, one phototropin and one superpigment (Briggs et al. 1999; Briggs 2001; Carabelli et al. 2010). There are two forms of phytochrome: (1) Red-light (λmax = 660 nm) absorbing form Pr, also known as physiological inactivation, with more stability; (2) Far-red-light (λmax = 730 nm) absorbing form Pfr. It belongs to physiological activation type and they can transform each other through chromophore isomerization (Gu et al. 1997). There are three main phytochrome (PHYA, PHYB, PHYC) in angiosperms, which are encoded by *PHYA*, *PHYB*, *PHYC* respectively; however, in dicotyledons, there are two additional phytochrome, PHYD and PHYE, which may be caused by gene replication events (Mathews et al. 2010). PHYD does not exist in tea plants (Mo et al. 2019). Photolabile PHYA mainly exists in etiolated tissues, and it is more sensitive to white light. Photolabile PHYB and PHYC mainly exist in the lighted tissue. PHYA and PHYB are mainly involved in photomorphogenesis and carotenoid synthesis of plant, they can also interact with basic helix-loop-helix (bHLH) transcription factors in a conformation specific manner to form phyinteracting interacting factors (PIFs, including PIF1-PIF5), which preferentially bind to many elements of light regulatory promoters, and then regulate the metabolism of pigment compounds (Moon et al. 2008; Quian-Ulloa et al. 2021). In response to low R: FR signal, the elongation and growth rate of stems and petioles are increased rapidly and significantly in many plants. However, it is often at the expense of the development of leaves and storage organs. These structural changes are accompanied by the increase of leaf angle (drooping) and apical dominance, resulting in the decrease of branching and tillering of dicotyledons. This reaction is collectively referred to as light avoidance syndrome (Casal et al. 1986; Holmes et al. 1997). This phenomenon can regulate the agronomic characters of tea plants with tender shoots as the main economic parts, and then affect the flavor and yield of tea. Phytochrome is modified by environmental signals, and there are some differences in the regulation mode of phytochrome gene under different light conditions, and the development of tea photosynthetic system can be changed. Therefore, it is speculated that using appropriate light composition to raise the leaf position to the unfiltered white light area can provide a basic survival strategy for the rapidly growing tea plant.

The close sensing of natural light environment is realized by the fusion of multiple signals (Møller et al. 2002). Photosensitive pigment signal transduction is a highly complex network occurring in multiple events, its optical properties are based on the interaction between dephenyl polypeptide chain and chromophore. PHY is synthesized in Pr form, after absorbing red light, the configuration of chromophore is changed, which leads to the change of the configuration and absorption spectrum of the dephenyl protein. Pr is transformed into the active form Pfr which plays an active role in most reactions. Pfr is irradiated by far red light and returned to the inactive state (Gu et al. 1997; Quail et al. 1991). Under the induction of red or white light, the stability of *PHYA* transcription products of monocotyledonous plants is reduced; the transcription products of dicotyledonous plants will not be significantly reduced under short-term red light irradiation, but continuous white light irradiation can reduce it. The expression levels of *PHYB* and *PHYC* in monocotyledons and dicotyledons are not affected by light; PHYA mainly plays a role in the high irradiance reaction under far red light and extremely low radiation, while PHYB and PHYC play a role in the low radiation and the transition between red light and far red light (Gu et al. 1997; Kong et al. 2018; Kreslavski et al. 2018; Park et al. 2018). In addition, photosynthetic pigments, chlorophyll and carotenoids in plants can also be used as photosensitive signals to absorb most of the visible light, although green light is reflected and converted (Franklin et al. 2005). Chlorophyll synthesis is regulated by light signal. Under different light quality conditions, the ratio of chlorophyll a/b is different. Yellow light and blue light are conducive to the synthesis of chlorophyll-b, and red light can promote the formation of chlorophyll-a (Zhou et al. 2006). Carotenoids and chlorophyll are components of photosynthesis and play a key role in plant biology, including daylighting, photo-oxidation quenching, and plant coloring. Among them, carotenoids are auxiliary pigments in tea plant photosynthesis, and it is the precursor of the terpene aroma components of tea (Kong et al. 2018; Wan 2008).

### Main factors affecting leaf color phenotype of photosensitive etiolated tea

The accumulation of pigments is the direct factor affecting the leaf color phenotype of photosensitive etiolated tea plant, including chlorophyll, carotenoids and flavonoids. The relative content, absolute content and distribution position of these pigments determine the leaf color of tea plant, and the decisive factor is genetic factor, which is mainly the result of the variation of nuclear gene and chloroplast gene or the regulation and expression of protein transport characteristics by different environmental media (Zhang et al. 2020). Chlorophyll is the most abundant tetrapyrrole compound in higher plants, mainly including chlorophyll-a of blue-green and chlorophyll-b of yellow-green; which are the main pigments of photosynthesis in plants, and can capture light energy and drive electrons to transfer to the reaction center, and convert and redirect light energy(Fromme et al. 2003; Tanaka et al. 2007). Carotenoids are a kind of polyisoprene pigment, which is a fat-soluble pigment embedded in chloroplast and chromosomal membrane, and showing mainly orange and yellow. Its composition is very conservative in plant classification, including α-carotene and β-carotene and xanthophylls (including β, ε-xanthophyll lutein, β, β-xanthophylls violaxanthin, neoxanthin, antheraxanthin and zeaxanthin), which are the determinants of the structure of photosystem I and II, what’s more, carotenoids can bind and stabilize photosynthetic complexes and improve the collection capacity of chlorophyll binding proteins. Therefore, they are also known as complementary pigments for chlorophyll to capture light energy and play an important role in the light protection of chloroplasts (Cazzaniga et al. 2012). Flavonoids, also known as anthocyanins, are mainly anthocyanins and xanthin. Their color changes with the change of acid–base environment, displaying red in acid environment and blue in alkaline conditions (Hu et al. 2014). The anthocyanins mainly affect the purple degree of tea leaves, while luteinin, myricetin and quercetin are yellow, which contribute to the yellow of leaves of tea plant (Fan 2019; Wang et al. 2017). The chloroplast of mesophyll cells in etiolated tea varieties is less and smaller, the number of grana lamellae is also very low, and the synthesis of photosynthetic pigment is also less than that in conventional green tea varieties. During the period of high light intensity in summer, the phenomenon of photoinhibition occurred, which led to the damage of chloroplast development and the formation of saccular vesicles, which gradually expand into vacuoles, resulting in the yellowing of leaf color. In the summer with the strongest light intensity, the total chlorophyll content and carotenoid content of yellowing tea plants are the lowest (Tian 2020; Xu 2016).

### Regulation of light on synthesis of main factors affecting leaf color phenotype of photosensitive etiolated tea plant

Light can affect the changes of pigment content and components from the level of plant cells, red light can up-regulate the synthesis of chlorophyll in peanut and promote the accumulation of carotenoids in tomato leaves (Han et al. 2018; Mengmeng et al. 2014). Blue light is beneficial to the accumulation of chlorophyll of hyacinth and up-regulates the carotenoids content of lettuce leaf (Anna et al. 2001). The pigment synthesis of cucumber and chrysanthemum seedlings treatment red-blue light is more than that treatment monochromatic light (Kim et al. 2012; Tang et al. 2011). When spinach is exposed to red-blue light, the synthesis of chlorophyll and carotenoids is up-regulated. It can be seen that light has obvious effects on plant pigment synthesis and leaf color phenotype (Huang et al. 2018). Different plants have different photoreceptors, so that their perception and expression patterns of different light sources are also different. The main reasons for the etiolated of “Huangjinya”, “Zhonghuang No. 1”, “Huangkui” and “Huangjinju” are the lack of chlorophyll and the differential accumulation of carotenoids (Li et al. 2016a, b). At present, the regulation of light on the metabolic pathway of these pigment compounds has been concerned and studied, and the mechanism of leaf color mutation of etiolated tea has been gradually solved.

**Fig. 1:**
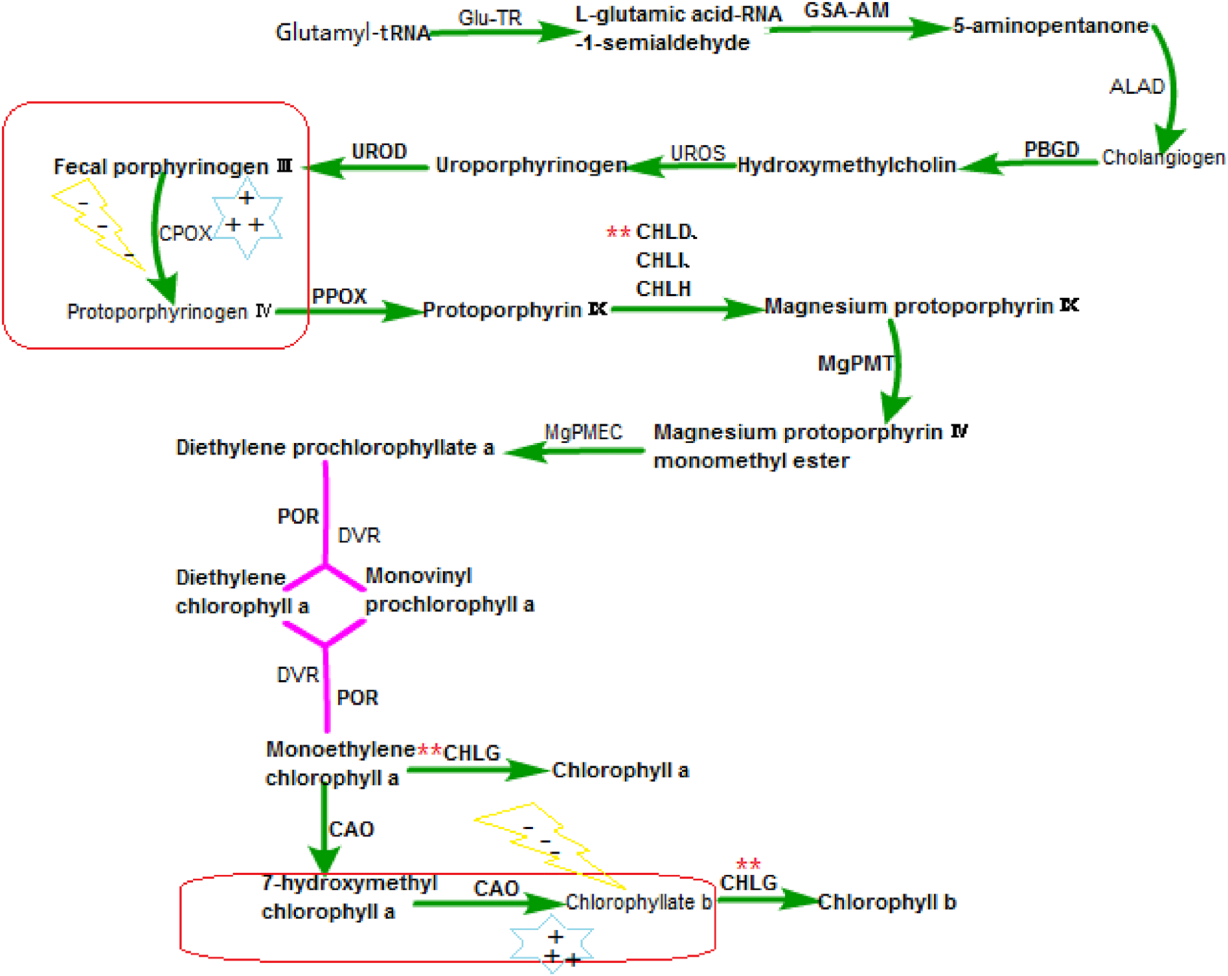
Synthesis pathway of chlorophyll (“**”: key enzymes or genes regulating chlorophyll synthesis; the red line frame is the blocking point of chlorophyll synthesis in etiolated tea that is exposed strong light. The related enzymes and related genes are shown in Table 1)

Chlorophyll synthesis is mediated by photoreceptors. Chlorophyll synthesis starts from glutamyl-tRNA (Fig. 1), and finally transforms into chlorophyll a/b. It is a set of strict and complex metabolic regulation, Chlorophyll synthesis can be divided into three processes: (1) Glu-tRNA to 5-aminopentanone (ALA); (2) AlA to magnesium protoporphyrin IX. The process is completed on the chloroplast matrix; (3) Magnesium protoporphyrin IX to chlorophyll a/b. In this processing, the synthesis of chlorophyll a/b occurs on the thylakoid membrane, and other reactions are completed on the chloroplast membrane (Block et al. 1980), in which 15 enzymes are required to participate in the catalysis (Table 1) (Ernesto Bianchetti et al. 2018; Gupta et al. 2014). So far, 27 genes encoding these enzymes have been isolated from Arabidopsis model plants (Beale 2005), among them, magnesium chelatase subunit D (CHLD) is the key enzyme, geranyl diphosphate reductase gene (*CHLP*) and chlorophyll synthase gene (*CHLG*) are key regulatory genes. During chlorophyll synthesis, the change of the coding gene of any enzyme may affect the characteristics of the enzyme, resulting in the inhibition of chlorophyll synthesis. The more forward the mutation position is, the more obvious the leaf color phenotype mutation will be. For example, when the mutation position is in the front, the green leaves can be changed into yellow or white. If the mutation occurs in the later stage, it will appear as spotted leaves, striped leaves, etc., moreover in the process of mutation, the phenomenon of leaf cell apoptosis may occur, which will threaten the life of the plant (Sakuraba et al. 2013). In general, the induction of light on chlorophyll synthesis is multidirectional, mainly in the following situations: (1) Strong light causes plants to produce photoinhibition, which inhibits the chlorophyll synthesis; (2) Moderate shading can increase the accumulation of chlorophyll, mainly as follows: under the condition of weak light or short shading, plants can make new adaptation response to slow down the stress of strong light, with the damage of chlorophyll reduced, and the content of chlorophyll-b and total chlorophyll increased, as well as the ratio of chlorophyll a/b decreased (Feng et al. 2019; Nyitrai et al. 1994; Shi et al. 2005); (3) The down-regulation of chlorophyll synthesis in plants under long-term insufficient light is mainly due to the reduction of the activity of key enzymes involved in plant photosynthesis and photosynthesis, resulting in the reduction of chlorophyll accumulation and the chlorosis of leaves; (4) Most plants cannot synthesize chlorophyll under dark conditions. These phenomena are mainly caused by the differential expression of genes regulating chlorophyll synthesis under different light intensities. During the greening of plants, *CHLH*, *CHLI1*, *CHLI2* and *CHLD*, which encode Mg-chelatase, can be induced by light (Stephenson et al. 2008). *CHLM*, which encodes Mg-protoporphyrin IX methyltransferase (MgPMT), is histochemically expressed in barley and tobacco, but its activity increased rapidly after several hours of light irradiation (Stenbaek et al. 2010). In addition, the early light induced-proteins increased rapidly under the stress of strong light, which reduced the activities CHLH and CHLI1subunits of Mg chelatase and GluTR, and reduced the synthesis of free chlorophyll (Tzvetkova-Chevolleau et al. 2007). Furthermore, light can also affect chlorophyll anabolism through metabolic signals between chloroplast and mitochondria. For example, strong light can inhibit cyanide-resistant respiration of mitochondria, making NADPH/NADP^+^ of plastids increase and promoting the protein interaction between Ferredoxin-NADP^+^ reductase (FNR) and translocon 62 (tic62) of chloroplast inner membrane, with the transport of chloroplast proteins to the membrane hindered, which result in the inhibition of the transport of chloroplast biosynthetic enzymes (Glu-TR, PBGD, PPOX, CHLH, PORB, CHLG) and geranylgeranyl reductase (GGR) to the membrane, which affecting the accumulation of chlorophyll in leaves (Zhang et al. 2015). In response to low light environment, genes related to pigment synthesis are differentially expressed in photosensitive etiolated tea plant. 89 differentially expressed genes (DEGs) have been obtained. More than 20% of DEGs are related to photosynthesis, porphyrin and chlorophyll metabolism, carotenoid biosynthesis pathway, which indicate that low light treatment is beneficial to photosynthesis and pigment metabolism of tea plants, and prolonged shading time (from 3 to 6 days), 12 genes related to light-harvesting complex are enriched in “photosynthesis antenna proteins” pathway. Under strong light, the expression of photosystem II (PSII) 10 kDa protein gene (*PsbR)* that relate to photosynthesis is inhibited, resulting in the destruction of PSII stability, as well as the development of chloroplast and the synthesis of chlorophyll are threatened, when shading treatment is performed, the expression of *PsbR* is up-regulated, which can reduce the damage of photooxidation and restore the structure of chloroplast (Liu et al. 2009; Suorsa et al. 2006; Wu et al. 2016). In chlorophyll synthesis pathway, *POR, ChlP*, which encode protochlorophyllide oxidoreductase (POR) and geranylgeranyl reductase respectively, are easily induced by strong light, affecting chlorophyll synthesis and photosensitivity. In the weak light, the expression of *ChlP* is down-regulated, while *POR* is up-regulated and chlorophyll accumulation has been increased (Dong et al. 2018). In the *PORC* albino mutant of Arabidopsis, the content of chlorophyll decreases dramatically, while the overexpression of *AtPORC* has the characteristics of tolerance to photooxidation damage. It has been reported that the regulation of light on chloroplast development and chlorophyll synthesis in tea plant is similar to that in Arabidopsis. These results suggest that the inhibition of *POR* expression may be an important reason for the etiolated leaf phenotype of tea plants that are suffered from strong light (Pattanayak et al. 2011; Tatsuru et al. 2003; Wu et al. 2016; Zhou et al. 2013). Another reason for the chlorosis of tea leaves is that the expression of *STAY-GREEN SGR*, a gene involved in chlorophyll degradation, is up-regulated, which accelerate the degradation of chlorophyll (Ma et al. 2018).

**Table 1:** Genes and enzymes involved in chlorophyll biosynthesis in plant

Code	Enzyme	Abbreviation	Gene	Gene annotation
1	Glutamyl-tRNA reductase	GluTR	*HEMA1*	At1g58290
			*HEMA2*	At1g09940
			*HEMA3*	At2g31250
2	Glutamate-1-semialdehyde-2,1-aminomutase	GSA-AM	*GSA1*	At5g63570
			*GSA2*	At3g48730
3	δ-Aminolevulinic acid dehydratase	ALAD	*HEMB1*	At1g69740
			*HEMB2*	At1g44318
4	Porphobilinogen deaminase	PBGD	*HEMC*	At5g08280
5	Uroporphyrinogen III synthase	UROS	*HEMD*	At2g26540
6	Uroporphyrinogen III decarboxylase	UROD	*HEME1*	At2g40490
			*HEME2*	At3g14930
7	Coproporphyrinogen III oxidase	CPOX	*HEMF1*	At1g03475
			*HEMF2*	At4g03205
8	Protoporphyrinogen IX oxidase	PPOX	*HEMG1*	At5g14220
			*HEMG2*	At4g01690
9	Magnesium chelatase H subunit	CHLH (GUN5)	*CHLH*	At5g13630
	Magnesium chelatase I subunit	CHLI	*CHLI1*	At4g18490
	Magnesium chelatase D subunit	CHLD	*CHLI2*	At5g45930
10	SAM Mg-protoporphyrin IX methyltransferase	MgPMT	*CHLM*	At4g25080
11	Mg-Proto IX monomethyl ester cyclase	MgPMEC	*CRD1*	At3g56940
12	3,8-divinyl Chlide 8-vinyl reductase	DVR	*DVR*	AT5g18660
13	NADPH: protochlorophyllide oxidoreductase	POR	*PORA*	At5g54190
			*PORB*	At4g27440
			*PORC*	At1g03630
14	Chlorophyllide a oxygenase	CAO	*CAO*	At1g44446
15	Chlorophyll synthase	CHLG	*CHLG*	At3g51820

Some studies have shown that genes light-harvesting complex II chlorophy a/b binding protein (LHCII, CSA035910) and STAY-GREEN (SGR, CSA024979) who related to chlorophyll biosynthesis and protein network with neural precursor cell expressed developmentally downregulated 8 (NEDD8) as the core may also be factors affecting the color change of etiolated tea leaves (Ma et al. 2018). Under strong light, there are 2 blocking points of chlorophyll synthesis in leaves of “Huangjinya”: (1) Fecal porphyrinogen III (COPPIII) to Protoporphyrin IX (PPIX), which is the transition from the second process to the third process of chlorophyll synthesis, and the synthesis place is shifted; (2) Pchlide to chlorophllide b, in this process, the protein and gene expression of POR and chlorophyllide a oxidase (CAO) are significantly also down-regulated by strong light (Fan 2019; Yangen et al. 2019). Compared with shading tea leaves, the expression of pheochlorophyllate a monooxygenase gene and protein related to the degradation of prochlorophyllate-a are up-regulated in etiolated tea leaves which expose normal light, which reduce the accumulation of chlorophyll a/b (Fan 2019). In fact, in addition to the accumulation of pigment compounds, the transformation mechanism of other secondary metabolites in etiolated mutation tea has also made a new adjustment, for example, albino tea plants lacking photosynthetic pigments will accumulate more flavonoids, especially ortho-dihydroxylated B-rings, and this phenomenon will also react on leaf color. However, the current genetic transformation system of photosensitive yellowing tea is not clear, so the transformation mechanism of photosensitive yellowing tea is not clear. Therefore the other effects caused by tea leaf color mutation and the interaction mechanism between the effects need to be further studied. MYB family also take part in pigment biosynthesis of light-mediated, which affects the accumulation of pigment when plant is in a state of light stress, and this argument has been confirmed in the physiological response mechanism of arabidopsis albino mutant (Quaedvlieg et al. 1996) and litchi (Li et al. 2013) responding to light. After shading treatment, MYB family also show differential expression (up-regulated or down-regulated) in etiolated tea plant (Wu et al. 2016), therefore, it is speculated that MYB transcription factors may play an important role in the process of formation of leaf color. What's more, the high expression levels of *CsPHYA* and *CsPHYB* can promote the accumulation of chlorophyll, and phytoene synthase (PSY) silencing can also destroy chloroplast organs and chlorophyll levels, thereby reducing photosynthetic efficiency, which have an important impact on the “green” accumulation of etiolated tea leaves with exposed red light (Liu et al. 2014; Tian et al. 2019). The thylakoid development of etiolated tea varieties is good, but strong sunlight inhibited the development of chloroplast, which lead to the poor development of chloroplast, and then restrain the accumulation of grana and the development of thylakoid. What’s interesting is that development of chloroplast can be quantified by reducing the light intensity appropriately, and then the abundance of chloroplast, the ultrastructure of thylakoid membrane system and the accumulation of grana can be induced to change in favor of chlorophyll synthesis and accumulation, while this physiological stress change is the self-protection mechanism of tea plant (Jiang et al. 2020; Liu et al. 2017, 2018).

**Fig. 2:**
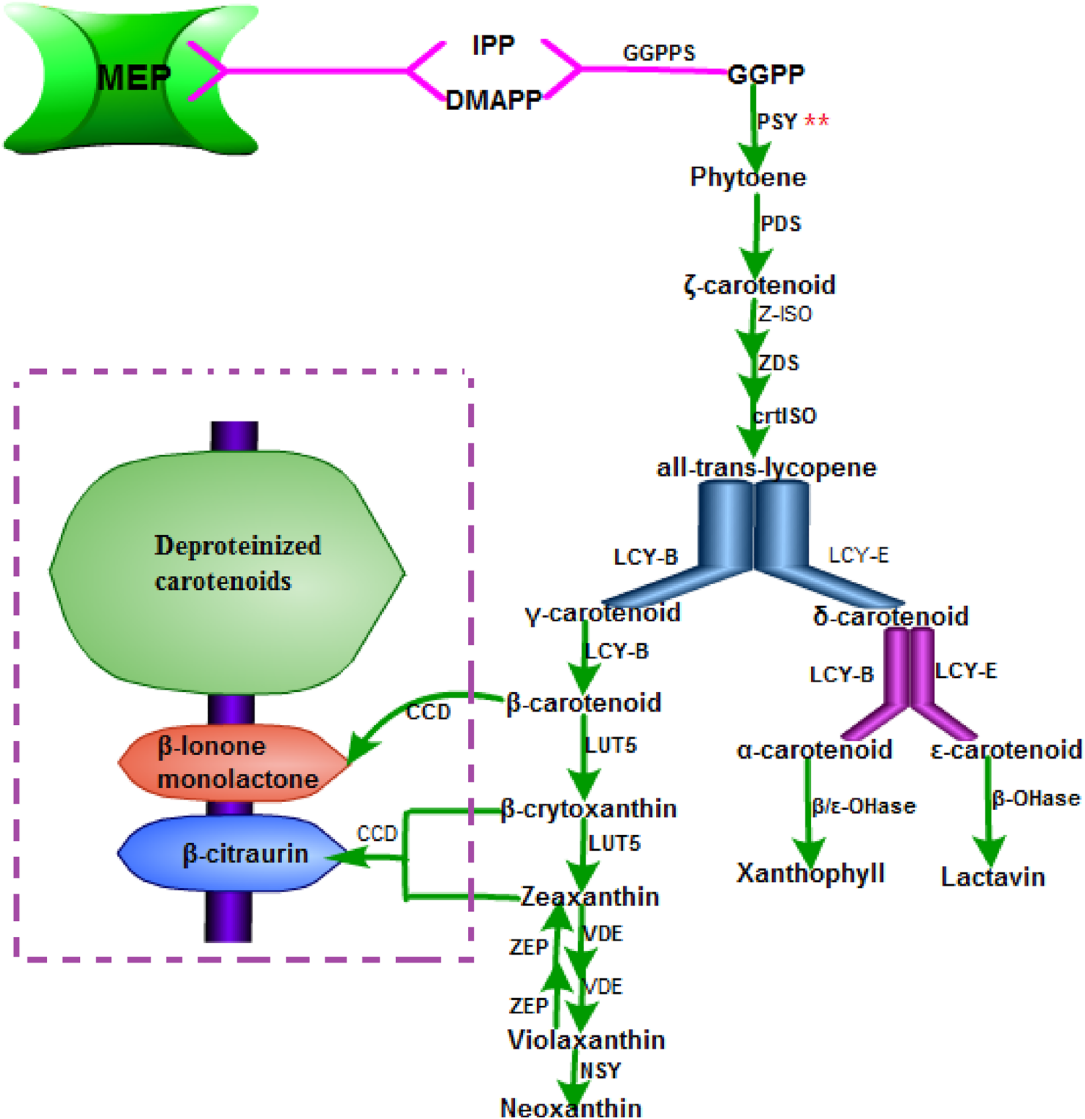
Synthetic pathway of carotenoids (“**”: the key enzymes or genes that regulate carotenoid synthesis, and the dotted box is the degradation of carotenoids. The related enzymes and genes are shown in Table 2)

Plant carotenoids are a group of pigment family with colors ranging from yellow to red. They are related to light collection and can reduce the damage caused by excessive light to plants. Except chlorophyll, all photosynthetic pigment-protein complexes that catalyze light collection and electron transfer are bound to carotenoids (Telfer 2005). The site of carotenoid synthesis is chloroplast. Carotenoids synthesis starts from the condensation of isomers of isopentenyl diphosphate (IPP) and dimethylallyl diphosphate (DMAPP), and then geranyl diphosphate (GGPP) is synthesized through the catalysis of geranyl diphosphate synthese (GGPPS), afterwards, two GGPPs are catalysised by PSY, phytoene desaturase (PDS) and ζ-carotene isomerase (Z-ISO), ξ-Carotene desaturase (ZDS) and carotenoid isomerase (crtISO) to produce lycopene, that is, the first carotenoid is synthesized. The synthesis of phytoene is the most strictly regulated link in the whole carotenoid synthesis process. PSY is the first and key enzyme in this process. The transcription and regulation of the gene encoding this enzyme is the main driving force of carotenoid synthesis (Quian-Ulloa et al. 2021; Toledo-Ortiz et al. 2010; Zhang et al. 2020), which are shown in Fig. 2. The precusor of carotenoids synthesis is provided by methyl erythritol-4-phosphate pathway (MEP) (Rodríguez-Villalón et al. 2009).

The synthesis of carotenoids is also induced by light environment, and different light environments have different effects on its synthesis and regulation. Under strong light conditions, high level expression of carotenoid ε-hydroxylase (LUT1) can effectively promote the accumulation of lutein and other compounds with non-photochemical quenching (NPQ), which has important influence in protecting the leaves of etiolated tea plants from excessive heat caused by strong light. Zeaxanthin can decompose the excessive chlorophyll excited state in the photosystem and scavenge oxygen free radicals (DemmigAdams et al. 1996; Jahns et al. 2012; Kawabata et al. 2014). High level expression of *LCYB*、zeaxanthin epoxidase gene (*ZEP*) and violaxanthin deepoxidase gene (*VDE*) promote the cycle and accumulation of violaxanthin and lutein, and these two compounds are beneficial to dissipate energy of etiolated tea leaves under high sunlight intensity, as well as acting as light protection for PSII. *VDE* can affect the differential expression of etiolated tea leaves under shading and light conditions, but the accumulation of lutein that is regulated by *VDE* in carotenoids metabolic pathway in the two environments is basically the same, suggesting that *VDE* and lutein are not the key factors affecting tea leaf color (Jahns et al. 2012; Wu et al. 2016). Under the condition of white light, PHYB is activated to make HY5 released from COP1/DDB1/CUL4 complex, at this same, the PHYB phosphorylates PIFS and causing it to be degraded by 26S proteasome, so HY5 is accumulated, which can bind to LRE (e.g., E and G-boxes) in PSY promoter, and carotenoid synthesis is induced (Quian-Ulloa et al. 2021; Rausenberger et al. 2010). In the shade condition (lower R/FR), PHYA is activated, which perform the work pathway of PHYB, and the synthesis of PSY and carotenoids in chloroplast are decreased. In addition, by CHLP-seq and RNA-seq identification, it is found that PSY is related to PHYA regulation. In contrast, the synthesis and regulation of carotenoids in the shade condition is more complex, and the process is closely related to the photomorphogenesis of light. In particular, carotenoids are involved in energy transfer, in which they play an important role for scavenging of singlet oxygen in photosynthetic reaction center (Cazzaniga et al. 2012; Telfer 2005). The mechanism of shading on plant secondary metabolism and photomorphogenesis is specific and complex, which is related to various photoreceptors and light signal transduction (Sano et al. 2018). Shading inhibited the expression of flavonoid biosynthesis related genes and induced the expression of carotenoid biosynthesis related genes, and the biosynthetic pathway of carotenoids and flavonoids makes a difference in the discoloration mechanism of tea leaves. The expression of most genes related to carotenoid biosynthesis increased after moderate shading, while all genes related to carotenoid biosynthesis are down-regulated in strong light (Li et al. 2016a, b; Quian-Ulloa et al. 2021; Song et al. 2017). In the dark environment, the photoreceptors are not active in the cytoplasm, and PIFS are fixed by de-etiolated (DET1)/damaged DNA binding protein 1 (DDB1)/CUK4 complex, which makes PIFS been bound the LRE of binding to PSY promoter and PIFS are inhibited, resulting in the inhibition of carotenoid synthesis. Constitutively photomorphogenic1 (COP1)/DDB1/cullin 4 (CUL4) complex can directly binds and trigger degradation of the basic helix-loop-helix (bHLH) HY5 and HFR1 transcription factors which have positive effects on light signal to make them degradation, which indicates that HY5 has antagonistic effect on PIF1 in the process of light morphogenesis, therefore, it suggests that HY5 has a positive effect on the accumulation of carotenoids, what’s more, the transcription factors of PIFs down- regulate carotenoid accumulation by specifically inhibiting the encoding *PSY* (Quian-Ulloa et al. 2021; Toledo-Ortiz et al. 2010). In actual production, carotenoid accumulation is affected by three pathways: synthesis, degradation and storage, no matter which pathway is changed, the color of leaves can be affected (Table 2).

**Table 2:** Genes and enzymes involved in carotenoids biosynthesis in plant

Code	Enzyme	Abbreviation	Gene	Gene annotation
1	Phytoene synthase	PSY	*AtPSY*	AT5G17230
2	Phytoene desaturase	PDS	*AtPDS3*	AT4G14210
3	*ζ-carotene isomerase*	Z-ISO	*Z-ISO*	AT1G10830
4	ξ-Carotene desaturase	ZDS	*AtZDS*	AT3G04870
5	Carotenoid isomerase	crtISO	*CRTISO*	AT1G06820
6	Lycopene β-cyclase	LCY-B	*AtLYC*	AT3G10230
7	Lycopene ε-cyclase	LCY-E	*AtLUT2*	AT5G57030
8	β/ε-Carotene Hydroxylase	β/ε-OHase	*DSM2*	Q10SE7
9	Carotenoid ε-hydroxylase	LUT1	*AtLUT1*	AT3G53130
10	Carotenoid β- hydroxylase	LUT5	*AtLUT5*	AT1G31800
11	Zeaxanthin epoxidase	ZEP	*AtABA1*	AT5G67030
12	Violaxanthin deepoxidase	VDE	*NPQ1*	AT1G08550
13	Neoxanthin synthase	NSY	*NSY*	SLY543649
14	9-*cis*-epoxycarotenoid dioxygenase	NCED	*NCED2*	AT4G18350
			*NCED3*	AT3G14440
			*NCED4*	AT4G19170
			*NCED5*	AT1G30100
			*NCED6*	AT3G24220
			*NCED9*	AT1G78390
15	Carotenoid cleavage dioxygenase	CCD7	*CCD7*	AT2G44990
		CCD8	*CCD8*	AT4G32810

Anthocyanins are water-soluble pigments, which are mainly concentrated in the vacuoles of the subcutaneous cell layer of vegetative tissues, and most of them are in the form of glycosides in tea plant, in addition, they can absorb potentially destructive UV-B radiation and protect plants (Hoch et al. 2001; Mei et al. 2021). The tea varieties “Zijuan” and “Ziyan” with high anthocyanin content belong to specific tea resources, which have buds and leaves of red–purple all year round. Anthocyanin synthesis is a branch of plant flavonoid synthesis pathway, which is an extension of phenylalanine and flavonoid pathway, beginning with aminolysis reaction catalyzed by phenylalanine ammonialyase (Mei et al. 2021). Light intensity or light quality conditions affect anthocyanin synthesis (Mancinelli et al. 1974; Wei et al. 2016). Strong light can induce up-regulation of genes encoding chalcone synthase (CHS), dihydroflavonol reductase (DFR), anthocyanin synthase (ANS) and anthocyanin reductase (ANR) in anthocyanin biosynthesis pathway, so as to increase accumulation of anthocyanins. Furthermore, the expression of *CsAN1*, *CsGL* and *CsEG*L, which regulate anthocyanin synthesis, is high with expending lighting time, which promotes the expression of MBW complex and is beneficial to anthocyanin accumulation (Li 2014; Sun 2016). The concentration of anthocyanins in tea plant mainly affects the phenotype of purple bud and leaf of tea plant (Nesi et al. 2001; Shen et al. 2018). The leaf color phenotype of photosensitive etiolated tea resources belongs to etiolated mutant, which does not belong to variation tea strains of anthocyanin metabolism regulation. The color characteristics of anthocyanins also suggest that the yellow phenotype leaf color has little relationship with anthocyanin specific accumulation. Hence, the regulation of light on anthocyanins is not our main task.

### Differential accumulation of key factors of leaf color of photosensitive etiolated tea in different light environment

Leaf color is an important index to measure the quality of tea (Taylor et al. 2010). There is a significant correlation between the degree etiolated and the light intensity of the photosensitive tea. The special color of this mutant can be regarded as a new property with commercial value. When the light intensity exceeds the light intensity threshold, it is easy to cause the bud burned and damaged (Li et al. 2016a, b; Tian 2020). The research status of photosensitive etiolated tea is shown in Table 3.

**Table 3:** Research progress of key pigment compounds of photosensitive etiolation tea plant

No.	Main research contents	References
1	The discoloration mechanism of leaf color in response to different light signals is studied at the molecular level	Tian et al. (2021); Du (2009); Song et al. (2017)
2	Different omics techniques are used to compare the molecular mechanism and metabolism of pigment compounds in etiolated tea in response to light intensity	Fan (2019)
3	Non-targeted metabonomics is used to analyze the causes of color variation of tender shoots of etiolation tea plants	Li et al. (2016a; b)
4	Transcriptome sequencing, ultrastructural analysis and biochemical analysis are used to analyze the causes of leaf color variation and the differential accumulation of pigments in etiolated tea plants	Zheng et al. (2021); Hao et al. (2020); Jiang et al. (2020); Liu et al. (2017); Wang et al. (2014); Yamashita et al. (2021)
5	Effects of light intensity on transcription of genes related to chlorophyll and carotenoid biosynthesis and chloroplast ultrastructure of etiolated tea plants	Li et al. (2016a; b)
6	Effects of shading on tea pigment compounds, leaf color and photoreceptors	Yangen et al. (2019); Sano et al. (2018); Tian et al. (2019)
7	Physiological characteristics and mechanism of leaf color response to light quality in etiolated tea plant	Tian (2020)
8	De novo sequencing of transcriptome was used to analyze the complex light response regulatory network of etiolated tea plant	Wu et al. (2016)
9	Photosynthetic characteristics and chloroplast ultrastructure of tea resources with different leaf colors	Xu (2016)

The reason for the yellow phenotype of tea leaves in “Baijiguan” and other tea resources is the lack of chlorophyll and carotenoid content, which makes them more vulnerable to UV stress (even under normal light conditions) (Wu et al. 2016; Zheng et al. 2021). In recent years, the expression characteristics of phenotypic mutants of bud and leaf color have been gradually reported. Under natural sunlight, the transcription level of genes regulating biosynthesis of carotenoid and chlorophyll in “Huangjinya” is lower than that in “Fudingdabai” (control), and most of the genes are up-regulated by the increase of medium shading, which indicate that strong light is not conducive to the biosynthesis of carotenoids and chlorophyll precursors in “Huangjinya” (Li et al. 2016a, b). The chloroplast structure in the leaves of etiolated tea is in incomplete state, the basal grains and thylakoid structure are disordered under the electron microscope. The structure of the chloroplast in the disordered state can be repaired by shading treatment (Du 2009). Under different wavelength light treatment, the color of “Huangjinya” leaves will change, which display that white and blue light can effectively keep the yellow of leaves, while red light makes the leaves of “Huangjinya” green. The contents of chlorophyll-a and chlorophyll-b in the second leaf of Huangjinya treated with red light (610–720 nm) increase by 1.54 and 2.44 times, respectively, and compared with white light treatment, the effects of blue light treatment on the contents of chlorophyll and carotenoids in the second leaf were not obvious (Tian et al. 2021). One hypothesis of the effect of monochromatic light on the leaf color of “Huangjinya” is that red light promotes the synthesis of chlorophyll (Ernesto Bianchetti et al. 2018; Gupta et al. 2014). The synthesis of chlorophyll is mediated by photoreceptors. Due to the different light harvesting efficiency of different photosynthetic pigments, the light quality has a great influence on the photochemical reaction (Hoffmann et al. 2015; Xu 2016). However, the effect of different spectral conditions on the photomorphogenesis of “Huangjinya” mainly show that the NPQ value of “Huangjinya” grown in red light environment and blue light (400–510 nm) is higher than that in white light, indicating that higher energy consumption and photoinhibition are needed to protect PSII from damage (Hoffmann et al. 2015). In the UV environment, the expression of three glutamyl-tRNA reductase genes (*HEMA*), four *POR*, four uroporphyrinoden decarboxylase genes (*UROD*) and five magnesium chelatase genes (*CHL*) that are related to chlorophyll synthesis are down-regulated, and the expression one *PDS*, two *ZDS*, one *VDE*, six *ZEP*, one Lycopene beta-cyclase genes (*LCYB*), and one Lycopene epsilon-cycylase genes (*LCYE*), regulating carotenoid biosynthesis pathway, are also down-regulated (Tian 2020). In addition, the expression of genes regulating the photosystem of etiolated tea plants is great difference in UV treatment, among the significantly down-regulated genes, most of them are related to the light harvesting pigment complex, among them, light capture complex II chlorophyll a/b binding protein 1 gene (LHCB1: TEA030366.1) and light capture complex I chlorophyll a/b binding protein 4 gene (LHCA4: TEA008208.1) are higher, in this treatment, five phytochrome genes (*PHY*) and cryptochrome genes (*CRY*) are also down-regulated, thereinto, *PHYB2*(TEA031363.1) is down-regulated the most in *PHY*, followed by *PHYE* (TEA001678.1) and *PHYA1* (TEA002223.1), and *CRY1a* (TEA012558.1) is down-regulated the most in *CRY*, showing no expression. CRY1b (TEA023230.1) and CRY2 (TEA009050.1) are down-regulated 9.53 and 2.66 folds, respectively. Catechins are a class of flavonoids, which have a significant correlation with the content of chlorophyll in tea, and the level of catechins is also affected by environmental factors (John et al. 2003; Premkumar et al. 2008; Wei et al. 2011), especially light. However, the mechanism of metabolic correlation between the two is unclear. Some scholars inferred that light activated flavonoid biosynthesis might be a stress response to protect tea plants from UV damage caused by sunlight (Agati et al. 2012). This may be one of the reasons that tea plants accumulate a lot of flavonoids, and it also shows that it is related to leaf color. PSY silencing can also damage chloroplast organs and reduce chlorophyll level, thereby reducing photosynthetic efficiency (Liu et al. 2014). In general, the contents of carotenoids and chlorophyll in photosensitive etiolated tea plants are low, but the levels of them can be increased by shading. Most genes involved in carotenoid biosynthesis are up-regulated after moderate shading, and these genes are photosensitive.

## Conclusions

This paper reviews the important research progress of the expression of key factors affecting the leaf color of photosensitive etiolated tea plant under different light conditions. In this work, the regulation of light on the synthesis of pigment compounds in etiolated tea is state of the art. Nevertheless, the photosensitive signal in tea plant is mainly based on the research conclusion of arabidopsis. At present, direct evidence has been obtained for the response of light conditions in the photoperiod to regulate pigment synthesis in tea plants. At present, direct evidence has been obtained that photoetiolated tea plants respond to light conditions to regulate pigment synthesis, and its mechanism has been partially elucidated. In addition, the different accumulation of pigment compounds under different light sources and its mechanism are reviewed. As an excellent mutant tea resource, photosensitive etiolated tea plant can be used to produce green tea that caters to the taste preference of market consumers. However, its leaf color is regulated by illumination intensity, with high photosensitivity. Strong light can easily reduce the stress resistance, photosynthetic capacity and chlorophyll content of leaves, resulting in leaf burned or color turned into green because of insufficient light. Based on the phenomenon of apparent response of tea plants to light stress, we fully understand the “management” of light on leaf color phenotype of etiolated mutant tea plant, which is helpful to solve the problem of how to reasonably maintain its physiological and biochemical characteristics without being damaged by light intensity, and then improve the yield of etiolated tea. Chlorophyll and carotenoids are accumulated in the leaves of plants with active photosynthesis, which are located in the light collection complex, and can affect the growth and development of tea plants, the accumulation of secondary metabolites and regulate the leaf color in response to light conditions. In the complex physiological and metabolic mechanism of tea plant, to fully understand and master the relationship between pigment compounds and leaf color phenotype and metabolites of influencing tea quality, to reverse the regulation of light conditions to manage the growth and development of tea plants. If this work is done, whether we can obtain satisfactory fresh leaf materials of tea plant can be a meaningful subject for further research. At present, the research based on the photosensitive signal of tea plant is still in its infancy. With the genome-wide of tea plants with different leaf shapes being deciphered, we can further study the genetic transformation system of tea plants in the future, explore the photosensitive signal system of tea plants with different color phenotype, and improve our understanding of the light supplement system of tae plant. It can be more reasonable management of tea cultivation and production.

## Data Availability

Not applicable.

## Abbreviations

PHY A/B/C/D: Phytochrome A/B/C/D/E
*PHYA/B/C/D/E*: Phytochrome A/B/C/D/E gene
bHLH: Basic helix-loop-helix
PIFs: Phyinteracting interacting factors
Glu-tRNA: Glutamyl-tRNA
ALA: 5-Aminopentanone
CHLD: Magnesium chelatase subunit D
CHLH: Magnesium chelatase subunit H
*CHLI*: Magnesium chelatase I subunit gene
*CHLH*: Magnesium chelatase H subunit gene
*CHLP*: Geranyl diphosphate reductase gene
*CHLG*: Chlorophyll synthase gene
*CHLM*: SAM Mg-protoporphyrin IX methyltransferase gene
MgPMT: Mg-protoporphyrin IX methyltransferase
FNR: Ferredoxin-NADP^+^ reductase
PBGD: Porphobilinogen deaminase
PPOX: Protoporphyrinogen IX oxidase
PORB: Protochlorophyllide oxidoreductase
CHLG: Chlorophyll synthase
GGR: Geranylgeranyl reductase
DEGs: Differentially expressed genes
KEGG: Kyoto Encyclopedia of Genes and Genomes
PSII: Photosystem II
*PsbR*: 10 KDa protein gene
POR: Protochlorophyllide oxidoreductase
*POR*: NADPH -protochlorophyllide oxidoreductase gene
*ChlP*: Geranylgeranyl reductase gene
PSY: Phytoene synthase
LHCII: Light-harvesting complex II
NEDD8: Developmentally downregulated 8
COPPIII: Fecal porphyrinogen III
PPIX: Protoporphyrin IX
CAO: Chlorophyllide a oxidase
MYB: V-myb avian myeloblastosis viral
IPP: Isopentenyl diphosphate
DMAPP: Dimethylallyl diphosphate
GGPP: Geranyl diphosphate
GGPPS: Geranyl diphosphate synthese
PDS: Phytoene desaturase
Z-ISO: ζ-Carotene isomerase
ZDS: ξ-Carotene desaturase
crtISO: Carotenoid isomerase
HY5: Long hypocotyies
LUT1: Carotenoid ε-hydroxylase
NPQ: Non-photochemical quenching
*ZEP*: Zeaxanthin epoxidase gene
*LCYB*: Lycopene β-cyclase
*VDE*: Violaxanthin deepoxidase gene
CHS: Chalcone synthase
DFR: Dihydroflavonol reductase
ANS: Anthocyanin synthase
*CsAN1*: Camellia sinensis anthocyanin synthase gene 1
*CsGL*: *Camellia sinensis* Glabra
*CsEG*L: *Camellia sinensis* Enhancer of glabra
MYB: Basic helix-loop-helix, and tryptophan aspartic-repeat
MBW: MYB-bHLH-WDR
WDR: Tryptophan aspartic-repeat
*HEMA*: Glutamyl-tRNA reductase genes
*UROD*: Uroporphyrinoden decarboxylase genes
*LCYE*: Lycopene epsilon-cycylase genes
*CRY*: Cryptochrome genes
DET 1: De-etiolated
COP 1: Constitutive photomorphogenic
DDB 1: Damaged DNA binding protein 1
CUL 4: Cullin 4
